# *Xenopus*: Driving the Discovery of Novel Genes in Patient Disease and Their Underlying Pathological Mechanisms Relevant for Organogenesis

**DOI:** 10.3389/fphys.2019.00953

**Published:** 2019-07-30

**Authors:** Woong Y. Hwang, Jonathan Marquez, Mustafa K. Khokha

**Affiliations:** Department of Pediatrics and Genetics, The Pediatric Genomics Discovery Program, Yale University School of Medicine, New Haven, CT, United States

**Keywords:** *Xenopus*, gene discovery, organogenesis, disease model, mechanism discovery, genetics of congenital malformations, birth defects

## Abstract

Frog model organisms have been appreciated for their utility in exploring physiological phenomena for nearly a century. Now, a vibrant community of biologists that utilize this model organism has poised *Xenopus* to serve as a high throughput vertebrate organism to model patient-driven genetic diseases. This has facilitated the investigation of effects of patient mutations on specific organs and signaling pathways. This approach promises a rapid investigation into novel mechanisms that disrupt normal organ morphology and function. Considering that many disease states are still interrogated *in vitro* to determine relevant biological processes for further study, the prospect of interrogating genetic disease in *Xenopus in vivo* is an attractive alternative. This model may more closely capture important aspects of the pathology under investigation such as cellular micro environments and local forces relevant to a specific organ’s development and homeostasis. This review aims to highlight recent methodological advances that allow investigation of genetic disease in organ-specific contexts in *Xenopus* as well as provide examples of how these methods have led to the identification of novel mechanisms and pathways important for understanding human disease.

## Introduction

The frog has served as a powerful tool for understanding human physiology dating back to early efforts in biomedical research. Many researchers found this model appealing due to its prevalence as a pregnancy test (Elkan, 1938). Injected human chorionic gonadotrophin induces ovulation and facilitates fertilization of a large number of embryos for experimentation. Subsequently, there has been an ongoing effort to develop methods to interrogate biology in the *Xenopus* embryo. Recently, next generation sequencing technologies have allowed researchers to rapidly amass a compendium of candidate gene variants that are putatively disease causing (Zaidi et al., 2013; Glessner et al., 2014; Homsy et al., 2015; Sifrim et al., 2016; Jin et al., 2017; Li et al., 2017; Manheimer et al., 2018). A major challenge is annotating these candidate genes with pathogenesis mechanisms. While statistical analysis of variants and computational approaches to predict mutational effect are necessary to identify putative disease-causing patient variants, many of the candidate genes have no known relevant biological function suggestive of its role in disease pathogenesis. Therefore, there is a pressing need for model systems to decipher these mechanisms. This is where patient driven gene discovery and disease modeling in *Xenopus* have proven fruitful.

## Screening and Evaluation of Patient Variants

*Xenopus* tadpoles develop most organs in just 3 days and the cell fate map for each organ system is well defined, so rapid phenotyping in knockout animals is possible (Moody, 1987). In fact, several gene knockout *Xenopus* lines are available to researchers through the community resources Xenbase and the international *Xenopus* resource centers (Table 2). In addition, the ability to manipulate only one side of the embryos and use the un-injected side as an internal control by one of two cell injection strategy has rendered *Xenopus* as a useful model to decipher disease mechanisms of patient variants.

Also, advances in CRISPR/Cas9 technology allow screening genes for disease relevance rapidly and inexpensively. The efficiency of knockout through CRISPR/Cas9 targeting is sufficient for screening in the F0 population of *Xenopus* embryos (Blitz et al., 2013; Bhattacharya et al., 2015). These F0 mosaic knockouts can also be used as founders to establish mutant lines. Community created resources such as CRISPR Scan (Moreno-Mateos et al., 2015) which can facilitate targeting specific loci and avoiding off target effects (Doench et al., 2014) have greatly simplified CRISPR based gene depletion experiments. Various tools are available to assess CRISPR genome editing efficiency, such as tracking of indels by decomposition (Etard et al., 2017). Off target effects can also be further evaluated by designing multiple non-overlapping sgRNAs to verify that multiple gene disruptions lead to similar phenotypes. Alternatively, complementary methods such as the use of morpholino oligos can similarly validate the phenotypic effects of CRISPR in F0 knockout screens. Subsequently, to test the specificity of targeted gene depletion strategies, human derived mRNA can be co-injected to rescue a mutated phenotype.

While results for CRISPR based knock-in technologies look promising in *Xenopus* (Aslan et al., 2017), knock-ins of human gene variants have not yet been fully utilized. On the other hand, to test patient variants for pathology, gene depletion followed by rescue with either wildtype human mRNA or patient variant mRNA has been effective and highly efficient (Braun et al., 2018; Kulkarni et al., 2018). Another limitation of the *Xenopus* model is a lack of antibodies available to detect *Xenopus* proteins, and there is an on-going concerted effort to produce monoclonal antibodies which will be freely shared with the *Xenopus* research community (personal communication D. Alfandari).

## Emerging Methods in Evaluating Effects of Genetic Manipulations in Organogenesis

### Cardiac Morphogenesis

**FIGURE 1:**
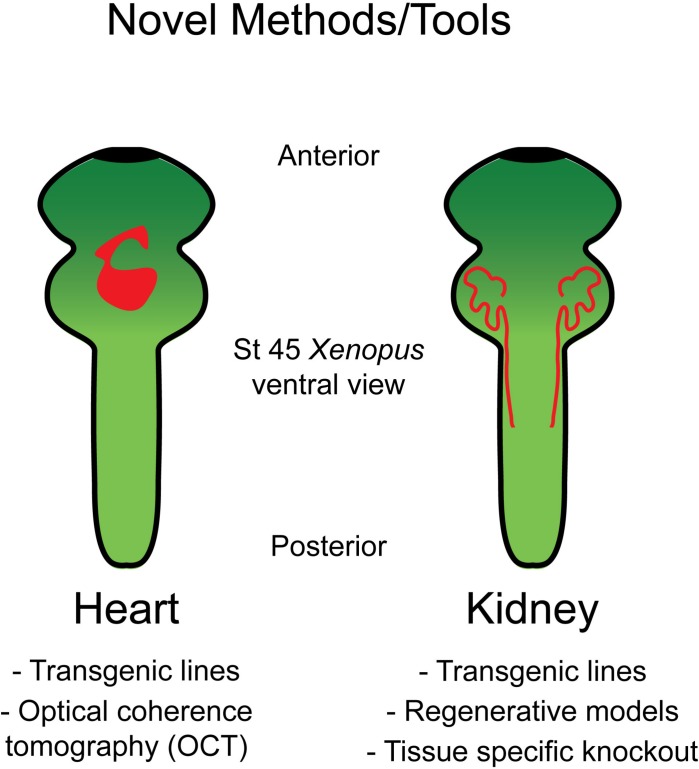
Tools in *Xenopus* allow for the study of heart and kidney development. Schematic of the organ systems in *Xenopus* along with available tools to interrogate these systems.

*Xenopus* is well suited for studying heart development as, unlike mice, *Xenopus* embryos do not require functional blood circulation for early cardiac development. This permits analysis of mutations that would prove embryonic lethal in mice. Additionally, the optical transparency which persists throughout early organogenesis enables assessment of morphological heart defects via multiple live imaging strategies (Figure 1).

Through targeted genome editing and a fate mapping, early heart developmental processes are well outlined in *Xenopus*. Similar to mammals, *Xenopus* heart development begins with pre-cardiac mesoderm formation during gastrulation (Sater and Jacobson, 1989). Cardiac precursor cells then migrate toward the ventral midline where they subsequently become specified as cardiac progenitor cells in two lineages: the first and second heart fields. These two heart fields undergo further remodeling to become two-atria/one ventricle and the outflow tract, respectively (Buckingham et al., 2005; Gessert and Kuhl, 2009). Several transgenic *Xenopus* lines have been engineered with fluorescent proteins fused to promotor regions of relevant cardiac markers to examine these developmental processes *in vivo*. One *Xenopus* line harbors the promoter of NKX2 fused to GFP and serves as a suitable marker for the early heart field (Sparrow et al., 2000), while MLC1v-GFP and SMAD3-GFP lines prove useful for labeling the myocardium and endocardium, respectively (Smith et al., 2005; Smith and Mohun, 2011; Table 1).

**TABLE 1:** Transgenic lines discussed in this review.

**Transgenic line**	**Development**	**Proposed utility**
Tg(nkx2.5:GFP)	Approx. 7.3 kb upstream of transcriptional start site of nkx2.5 (GU573788) fused to GFP. This region should contain the nkx2.5 promoter	Early heart field studies
Tg(mlc3:GFP)	8 kb of promoter of the *X. laevis* MLClv (myosin light chain 3) gene driving expression of EGFP (note MLClv synonym for = mlc3)	Myocardium studies
Tg(smad3:eGFP)	1.5 kb of promoter of the *X. laevis* smad3 gene driving expression of eGFP	Endocardium studies
Tg(WntREs:dEGFP)	7 copies of a TCF/LEF1 binding DNA element, a minimal TATA box and a reporter gene encoding destabilized eGFP and a polyA sequence	Wnt signaling in cardiogenesis
Tg(Dre.cdhl7:eGFP)	Approximately a 4.3 kb genomic fragment upstream of the Danio rerio cdhl7 driving expression of EGFP	Pronephric development

**TABLE 2:** A subset of mutant lines of interest to disease processes in the National *Xenopus* Resource Database.

**Organ of interest**	**Mutation**	**Species**	**Associated human disease**
**Kidney**	Pkhd1L	*X. Laevis*	Autosomal Recessive Polycystic Kidney Disease
	pkd2.L	*X. Laevis*	Polycystic Kidney Disease 2
	wdpcp.L	*X. Laevis*	Bardet-Biedl syndrome (ciliopathic genetic disorder that affects many parts of body including kidney failure)
	eya1.L	*X. Laevis*	Branchio-oto-renal syndrome
**Heart**	tbx5	*X. Tropicalis*	Hold-Oram syndrome (cardiac-limb syndrome)
	gdf1	*X.Tropicalis*	Double outlet right ventricle, tetralogy of Fallot, Right atrial isomerism
	Imna	*X. Tropicalis*	dilated cardiomyopathy
	myh6	*X.Tropicalis*	familial hypertrophic cardiomyopathy, dilated cardiomyopathy, atrial septal defect
			

Another exciting model is the Wnt reporter line developed in *Xenopus* (7X LEF/TCF-GFP) (Tran et al., 2010; Tran and Vleminckx, 2014; Table 1). Both canonical and non-canonical Wnt signaling is spatiotemporally controlled to orchestrate proper cardiac development (Nakamura et al., 2003; Naito et al., 2006; Tzahor, 2007; Ueno et al., 2007; Gessert and Kuhl, 2010). In the context of left-right patterning, Wnt and serotonin signaling were found to be crucial for left-right organizer (LRO) specification and differentiation (Nascone and Mercola, 1997; Beyer et al., 2012). In particular, Wnt direct target gene, *foxj1*, expression is up-regulated in ATP4a dependent manner that consequently controls motile ciliogenesis in the LRO (Walentek et al., 2012). Left–right patterning in turn has far reaching consequences for cardiogenesis (Reviewed in Duncan and Khokha, 2016; Garfinkel and Khokha, 2017). Wnt action during cardiogenesis can be divided into four stages. First, high levels of Wnt/β-catenin activity is required for the formation of prospective heart mesoderm (Smith and Howard, 1992; Antin et al., 1994). Once cardiac mesodermal cells colonize the ventral midline, Wnt signaling is damped down allowing for cardiac specification and generation of multi-potent progenitor cells (Marvin et al., 2001; Schneider and Mercola, 2001). Shortly thereafter, Wnt signaling is up-regulated for the expansion and proliferation of cardiogenic progenitor cells and repressed again for terminal differentiation of cardiomyocytes (Ai et al., 2007; Kwon et al., 2007). As the role of Wnt signaling in cardiac development is both dynamic and complex, further investigation is warranted in this *Xenopus* Wnt reporter line. Exemplifying the utility of this model, recent work using this Wnt transgenic *Xenopus* line has comprehensively delineated the spatial and temporal dynamics of Wnt signaling through whole-mount *in situ* hybridization and cross-sectioning of embryos (Borday et al., 2018).

Lastly, optical coherence tomography (OCT) has recently been employed as a reliable and efficient imaging modality to assess cardiac structural anomalies in *Xenopus* tadpoles (Deniz et al., 2017). OCT uses coherent light waves to capture cross-sectional images of tissues in live embryos (Kagemann et al., 2008). OCT can thus comprehensively measure *Xenopus* cardiac structures including the atria, trabeculated ventricle, atrioventricular valve, and the diameter of outflow tract. As evidence of its utility, depletion of the myosin heavy chain 6 (myh6) gene which has been shown to have variants that cause human cardiomyopathy (Abu-Daya et al., 2009) was employed in *Xenopus* embryos. OCT imaging of these embryos yielded successful dynamic assessment of cardiac defects such as dysregulated AV valve excursion times (Deniz et al., 2017). Overall, OCT and cardiac fluorescent transgenic models maximize the power of the optical transparency in *Xenopus* embryos by allowing for dynamic live imaging and observation of cardiac development at both cellular and sub-cellular levels.

### Kidney Morphogenesis

The embryonic kidney in *Xenopus* consists of a pronephros that is simplistic compared to human metanephroi (Fox and Hamilton, 1964; Vize et al., 1995); however, this provides a straightforward structure that can be readily interrogated (Figure 1). Additionally, regions of the pronephros correspond to regions of the human metanephros based on function and patterns of gene expression (Carroll and Vize, 1999; Carroll et al., 1999; Wild et al., 2000; Saulnier et al., 2002; Zhou and Vize, 2004; Alarcon et al., 2008; Raciti et al., 2008; Buisson et al., 2015). Although the whole mount *in situ* hybridization has been used to interrogate the pronephros based on expression, new tools allow kidney research to observe changes *in vivo*. Among these is a transgenic line with GFP fused to Cdh17 that facilitates visualization of the entire pronephros (Corkins et al., 2018; Table 1).

Recent efforts in kidney research in *Xenopus* have also yielded models of pronephric regeneration that show great promise (Caine and McLaughlin, 2013). These studies build upon work that has addressed pronephroi as *in vitro* explants (Moriya et al., 1993; Osafune et al., 2002; Asashima et al., 2009). As kidney tissue is susceptible to damage from genetic as well as acquired renal disease, understanding its regenerative potential may allow us to identify methods of recovering tissue function in the context of disease. Additionally, observing regeneration as opposed to organogenesis of the pronephros facilitates uncoupling signaling relevant for the generation of this specific organ from generalized processes of development. Such an approach may be essential for understanding the mechanisms of dysfunction observed due to patient-derived candidate gene variants.

The ability to target specific regions of the developing embryo is a powerful avenue to home in on factors relevant for specific pathways and tissues. Screening candidate genes based on patient variants in the *Xenopus* kidney is no exception. Although F0 CRISPR based knockout of genes is a powerful tool, this has been deployed largely in whole embryo approaches. Developing a tissue-specific targeting strategy is an important next step to screen for organ-specific dysfunction. Fortunately, this has been evaluated precisely in the context of the *Xenopus* pronephros wherein CRISPR/Cas9 was injected in a targeted manner to demonstrate that this technology could be applied in a subset of embryonic blastomeres that give rise to the pronephric tissue (DeLay et al., 2018). This advance shows that despite the mosaicism observed via the targeted use of CRISPR/Cas9 in the F0 generation, this technology can be used to observe downstream consequences of loss of function in particular regions of F0 embryos without the need to raise mutant lines. This may be particularly useful for studying the pronephroi in the context of gene knockout scenarios in which embryonic development is so severely affected as to preclude the study of later organ development. By adopting this approach to limit candidate gene screening to the embryonic kidney, we may be better equipped to answer what role a novel gene is playing in patient disease affecting this organ.

## Recent Patient Driven *Xenopus* Studies in Organogenesis

### Novel Mechanisms in Congenital Heart Disease (CHD)

Congenital heart disease (CHD) is the most prevalent class of birth defects leading to high infant mortality in the United States and yet for the vast majority of cases, the underlying molecular mechanisms remain elusive (Van der Linde et al., 2011; Triedman and Newburger, 2016). However, by coupling cost-effective sequencing technologies to gene editing tools in animal model systems, novel genetic variants from patients can be quickly analyzed *in vivo* in a high-throughput manner. This has been a productive approach in *Xenopus* as candidate genes have been efficiently analyzed for their functional cardiac relevance in *Xenopus* embryos.

For example, *RAPGEF5* which encodes a guanine nucleotide exchange factor for Rap-GTPase was found to have an internal duplication which would likely lead to a null allele in a heterotaxy patient. Depletion of Rapgef5 in *Xenopus* recapitulates the left-right patterning phenotype found in the patient. Unexpectedly, mechanistic studies established that RAPGEF5 regulates left-right patterning via Wnt signaling by regulating the nuclear localization of β-catenin (Griffin et al., 2018). As previously noted, Wnt signaling is critical for proper induction of dorsal mesoderm including cardiac precursors and the LRO. As such, dysregulation of Wnt signaling caused by Rapgef5 knockdown resulted in abnormal LRO formation and cardiac looping phenotypes. Moving beyond CHD, dysregulation of Wnt signaling is implicated in many human diseases especially colorectal cancers (Morin et al., 1997; Cancer Genome Atlas Network, 2012). Therefore, elucidating this transport machinery may lead to novel therapeutic targets for a host of diseases.

Another successful example of modeling heterotaxy candidate genes in *Xenopus* was demonstrated by identifying a novel functional role of inner-ring nucleoporins, Nup188 and Nup93 in the context of cilia. Nup188 and its interactant Nup93 were discovered to localize to the base of cilia suggesting a role outside of the nuclear envelope. Knockdown of these components results in the loss of cilia in the LRO subsequently leading to abnormal heart patterning (Del Viso et al., 2016). Cilia are key cellular structures necessary to generate and sense unidirectional fluid flow and induce asymmetric gene expression for proper organ situs (Wallingford and Mitchell, 2011; Boskovski et al., 2013; Blum et al., 2014; Yoshiba and Hamada, 2014). These studies demonstrate the complex genetic etiology of CHD and the successful use of *Xenopus* to identify and characterize disease mechanisms of pathogenic human variants.

### Novel Mechanisms in Congenital Disease of the Kidney

Work utilizing the developing *Xenopus* kidney has led to new roles and mechanisms for candidate genes in kidney development and disease. Congenital anomalies of the kidney and urinary tract (CAKUT) include a wide variety of patient presentations ranging from abnormal kidney and urinary tract size and morphology to tumor growth within this organ system (Rodriguez, 2014). As these anomalies comprise the most common cause of childhood renal failure, high throughout models for candidate CAKUT genes are useful for testing candidate genes and exploring pathogenesis mechanisms.

For example, variants of *NRIP1* were identified in patients with CAKUT (Vivante et al., 2017). These anomalies consisted of renal hypodysplasia and either vesicoureteral reflux or ectopia. Prior to this work, the mechanism of NRIP1 function in kidney development or even developmental at large was not established. This study also highlights the potential to investigate novel therapeutic pathways for patients in which variants are identified. Since retinoic acid signaling was downregulated by *NRIP1* mutation in this instance, manipulation of retinoic acid dependent pathways is an appealing avenue for therapeutic studies.

Studies of ciliopathies are another excellent example of these efforts. Though ciliopathies affect a wide array of organ systems including the heart (Duncan and Khokha, 2016), the kidney is particularly susceptible to the loss of function of cilia-related proteins. Ciliopathies are a diverse array of diseases caused by disrupted formation and/or function of cilia (Bisgrove and Yost, 2006; Gerdes et al., 2009; Oh and Katsanis, 2012; Werner and Mitchell, 2013; Choksi et al., 2014). Cilia are cellular extensions that have been shown to serve as mediators of extracellular signals in the case of primary cilia (Nachury and Mick, 2019) or a means by which cells coordinate extracellular fluid flow in the case of motile cilia (Mitchison and Valente, 2017). In the kidney, cilia dysfunction often manifests itself as a cystic change in tissue morphology that renders the kidney unable to regulate the urine concentration and hemofiltration (Ma et al., 2017). The mechanisms by which cilia dysfunction leads to this tissue level dysfunction are still not well understood.

In another example of patient driven gene discovery, identifying a role for the DNA repair protein NME3 in cilia established another example for a growing list of ciliopathies (Hoff et al., 2018). Depletion of NME3 resulted in renal malformations and left–right patterning defects typical of ciliopathies in *Xenopus* along with a loss of cilia in complementary vertebrate and cell culture models. The association of NME3 with the ciliary nephronophthisis proteins NEK8, CEP164, and ANKS6 supports its role as a ciliopathy gene. This discovery adds to a growing number of ciliopathy related genes that have known roles away from the cilium but seem to also play an important role when recruited to the cilium. This has far reaching implications for kidney disease biology, as the discovery of new roles and/or localizations for gene products will allow us to harness previous knowledge about these components to understand the disease. Complementarily, subsequent discovery of the proteins’ role in kidney morphogenesis may lead to understanding its function in other contexts. In the case of NME3, this work has led to a still evolving connection between primary cilia function and DNA damage repair, which may constitute an even broader pathway important for kidney development and homeostasis.

Similarly, recent studies have implicated mutations in several components of the outer-ring of the nuclear pore complex (NPC) in steroid resistant nephrotic syndrome (SRNS) (Braun et al., 2018). SRNS is a broad category of disease in which the body excessively excretes proteins in the urine thus leading to systemic fluid distribution imbalances and swelling which is most often linked to disruption of the renal vascular interface that functions in podocyte mediated fluid filtration (Dogra and Kaskel, 2017). Variants of outer-ring NUPs were tested in *Xenopus* knockdown models to verify their deleterious status (Braun et al., 2018). This study went on to determine that this effect on kidney morphogenesis is mediated through Cdc42 signaling important for filopodia (Nobes and Hall, 1995). These findings highlight that proteins essential for the function of every cell such as NPC components can still give tissue specific phenotypes suggesting that we have a great deal to learn about the pathogenesis mechanisms of human disease.

Though these studies demonstrate the power that patient driven gene discovery has for assigning disease causing variants in human kidney disease, they also begin to show how this approach aids in discovering novel mechanisms important for kidney morphogenesis and function. Moving forward, the many congenital kidney malformations that affect patients early in life may be an efficient source of genetic information to direct the study of kidney morphogenesis.

## Conclusion

Sequencing technologies continue to improve in speed, accuracy, and cost. Consequently, rather than phenotype driven therapy, patient genotype can be included in decision-making and treatment options tailored to individuals. However, one major challenge to this approach is determining the biological relevance of each variant as many candidate genes have no known role in disease. Therefore, each variant offers the opportunity for molecular function discovery in animal models. Studying organogenesis in *Xenopus* including that of the heart and kidney will allow us to unravel mechanisms of disease pathogenesis. Analysis of candidate genes in *Xenopus* will not only allow for assessing allele pathogenicity, but also promises to expand our understanding of developmental biology.

## Author Contributions

All authors wrote and edited the manuscript.

## Conflict of Interest Statement

The authors declare that the research was conducted in the absence of any commercial or financial relationships that could be construed as a potential conflict of interest.
